# Identifying dementia risk profiles for targeted interventions: A latent class analysis of at‐risk middle‐aged Australians

**DOI:** 10.1002/alz.70888

**Published:** 2025-11-07

**Authors:** Muhammad Rehan Sarwar, Amanda J. Cross, Kali Godbee, Gopisankar Mohanannair Geethadevi, Parker Magin, Mary Tullipan, Amanda L. Baker, Billie Bonevski, Stephanie A. Ward, Ajay Mahal, Vincent Versace, J. Simon Bell, Kevin Mc Namara, Sharleen L. O'Reilly, Dennis Thomas, Elizabeth Manias, Kaarin J. Anstey, Marlien Varnfield, Rajiv Jayasena, Rohan A. Elliott, Cik Y. Lee, Andrea Hernan, Denise van den Bosch, Catherine Ferreira, Johnson George

**Affiliations:** ^1^ Centre for Medicine Use and Safety Faculty of Pharmacy and Pharmaceutical Sciences Monash University Parkville Victoria Australia; ^2^ School of Medicine and Public Health The University of Newcastle Newcastle New South Wales Australia; ^3^ School of Population Health University of New South Wales Sydney New South Wales Australia; ^4^ National Drug and Alcohol Research Centre University of New South Wales Sydney New South Wales Australia; ^5^ Flinders Health and Medical Research Institute Flinders University Bedford Park South Australia Australia; ^6^ Centre for Healthy Brain Ageing University of New South Wales Sydney New South Wales Australia; ^7^ School of Public Health and Preventive Medicine Faculty of Medicine Nursing and Health Sciences Monash University St Kilda Victoria Australia; ^8^ Nossal Institute for Global Health University of Melbourne Melbourne Victoria Australia; ^9^ Deakin Rural Health Faculty of Health Deakin University Warrnambool Victoria Australia; ^10^ School of Agriculture and Food Science University College Dublin Dublin Ireland; ^11^ School of Exercise and Nutrition Science Deakin University Melbourne Victoria Australia; ^12^ Centre of Excellence in Treatable Traits College of Health Medicine and Wellbeing University of Newcastle Newcastle New South Wales Australia; ^13^ Asthma and Breathing Research Program Hunter Medical Research Institute Newcastle New South Wales Australia; ^14^ Monash Nursing and Midwifery Faculty of Medicine Nursing and Health Sciences Monash University Clayton Victoria Australia; ^15^ School of Psychology University of New South Wales Sydney New South Wales Australia; ^16^ UNSW Ageing Futures Institute University of New South Wales Sydney New South Wales Australia; ^17^ The Australian e‐Health Research Centre Health and Biosecurity CSIRO Herston Queensland Australia; ^18^ The Australian e‐Health Research Centre Health and Biosecurity CSIRO Parkville Victoria Australia; ^19^ Pharmacy Department Austin Health Heidelberg Victoria Australia; ^20^ Department of Nursing School of Health Sciences Faculty of Medicine Dentistry and Health Sciences The University of Melbourne Melbourne Victoria Australia; ^21^ North Western Melbourne Primary Health Network Docklands Victoria Australia

**Keywords:** ANU‐ADRI, dementia, latent class analysis, middle‐aged adults, modifiable risk factors, multidomain interventions, primary care

## Abstract

**INTRODUCTION:**

This study aimed to identify distinct dementia risk profiles in middle‐aged adults with two or more potential dementia risk factors, to inform targeted prevention strategies.

**METHODS:**

Cross‐sectional analysis of baseline sociodemographic, clinical, and dementia‐risk data from the HAPPI MIND trial. Dementia risk was assessed using the Australian National University Alzheimer's Disease Risk Index. Risk profiles were identified using latent class analysis (LCA).

**RESULTS:**

Among 403 participants (mean age 56.4 ± 5.7 years, 62.5% female), the median number of dementia risk factors was 5.0; hyperlipidaemia (92.5%), low cognitive activity (72.5%), obesity (57.6%), and hypertension (52.7%) were the most prevalent. Several risk factors showed significant positive correlations. LCA identified three distinct classes: *1−High Cardiometabolic Burden; 2−High Behavioural and Psychosocial Risk*; and *3−Low Risk with Healthy Behaviours*.

**DISCUSSION:**

The identified latent classes highlight heterogeneity of dementia risk profile in midlife. Tailored, multidomain interventions addressing each group's specific needs may improve dementia risk profiles and support broader health outcomes.

**Highlights:**

- Middle‐aged Australians who participated in the HAPPI MIND dementia risk reduction trial had a median of five modifiable risk factors.
- Significant positive correlations were observed between behavioral and clinical risk factors, such as depression, along with poor diet, social isolation, and smoking.
- Latent class analysis revealed three distinct profiles: *High Cardiometabolic Burden; High Behavioral and Psychosocial Risk;* and *Low Risk with Healthy Behaviors*.
- The findings suggest there is a need for personalized, multidomain prevention strategies tailored to individual risk profiles in primary care.

## 1 INTRODUCTION

Research into dementia has consistently highlighted a range of genetic, health, behavioral, and psychosocial factors that contribute to the onset risk.^1^, ^2^, ^3^, ^4^ However, the interplay among these risk factors, especially those that are modifiable and prevalent among middle‐aged adults, remains complex and not fully elucidated. Identifying how these risk factors cluster together can offer valuable insights into the epidemiology of dementia and pave the way for more targeted and personalized preventive interventions.

Middle age is a crucial period for addressing dementia risk factors, as lifestyle and health behaviors during these years have been shown to significantly impact cognitive decline and dementia risk later in life.^5^ *The 2024 Lancet Commission on Dementia Prevention, Intervention, and Care* estimated that up to 45% of dementia cases could be prevented or delayed by addressing modifiable risk factors across the life course, many of which are most impactful during midlife.^1^ Addressing potentially modifiable risk factors such as hyperlipidemia, hypertension, obesity, and low cognitive activity may provide an opportunity to reduce dementia incidence.^1^

Risk factors for dementia rarely exist in isolation; they often cluster within individuals, influencing both cognitive health and overall quality of life (QoL). Understanding how these risk factors interact and co‐occur is critical for designing effective, targeted, and prevention strategies. While most existing research has considered additive effects, showing that a higher number of risk factors is linked to greater risk of cognitive decline^6^, ^7^, ^8^ and dementia^9^, ^10^—this approach has key limitations. It fails to account for interrelationships among risk factors, does not consider their differential impact on outcomes, and overlooks distinct risk profiles within the population.

Latent class analysis (LCA) is a person‐centered analytical approach that captures population heterogeneity by identifying distinct subgroups based on shared characteristics. LCA has previously been used to uncover vascular risk profiles associated with dementia outcomes^11^, ^12^, ^13^ and to identify patterns of modifiable dementia risk factors among adults aged 60–64 years.^14^ This study is among the first to examine dementia risk profiles specifically in at‐risk middle‐aged adults. We aimed to address this gap by identifying distinct dementia risk profiles in a middle‐aged Australian population with two or more potential dementia risk factors, and examining their associations with cognitive health, cardiometabolic burden, and QoL. By identifying specific risk profiles in primary care and their relationships with health and lifestyle factors, this research seeks to offer insights into early prevention strategies that could help mitigate dementia risk and promote long‐term cognitive resilience.

## 2 METHODS

We conducted baseline data analyses of the HAPPI MIND (Holistic Approach in Primary care for PreventIng Memory Impairment aNd Dementia) trial, described in detail elsewhere.^5^ In brief, HAPPI MIND is a cluster‐randomized controlled trial of a multidomain behavior changes risk reduction intervention in at‐risk middle‐aged adults in New South Wales and Victoria, Australia. The intervention was led by primary care practice nurses. The target group was middle‐aged adults (45–65 years) with at least two dementia risk factors. Outcomes of interest included change in dementia risk assessed by the ANU‐ADRI (Australian National University Alzheimer's Disease risk Index), CAIDE (Cardiovascular risk factors Aging and Dementia risk score), and change in QoL utilizing the EQ‐5D‐5L instrument. The study was approved by the Monash University Human Research Ethics Committee (Project ID: 28273) and conducted in accordance with the ethical standards as laid down in the 1964 Declaration of Helsinki and its later amendments or comparable ethical standards.

**RESEARCH IN CONTEXT**

1. **Systematic review**: Various modifiable risk factors—such as depression, cardiometabolic conditions, and unhealthy behaviors—have been linked to cognitive decline. However, little is known about how these risk factors cluster within individuals and how the clusters relate to dementia risk in middle‐aged adults in primary care.
2. **Interpretation**: We identified three distinct dementia risk profiles among middle‐aged Australians with two or more potential dementia risk factors. The profiles varied by mental health status, cardiometabolic burden, and health behaviors, each showing unique patterns of dementia risk and quality of life outcomes.
3. **Future directions**: Understanding dementia risk as a multidimensional construct allows for tailored, multidomain prevention strategies. Interventions may be more targeted and effective when tailored to the specific risk profile of individuals.

### 2.1 Data collection/measurements

Data were collected by trained nurses. Participant demographic characteristics, including age, gender, country of birth, language spoken at home, education, employment, marital status, living arrangements, and family history of dementia, were collected via patient self‐report. Participant medical records were reviewed to obtain medical history.

Validated tools were used to collect participant data across multiple domains, with methods including self‐report questionnaires and clinician‐administered assessments (see Table 1 below). In particular, detailed information was collected for the main study outcomes:

***ANU‐ADRI*** ^15^— This is an evidence‐based and validated tool aimed at assessing individual exposure to risk and protective factors known to be associated with developing Alzheimer's disease. The range of scores is −18 to +79 (maximum +38 for adults aged < 65 years), with higher scores indicating greater dementia risk.
***CAIDE*** ^16^— A validated midlife dementia risk score that combines demographic, vascular, and lifestyle factors to estimate an individual's risk of developing dementia. Scores range from 0 to 15, with higher scores indicating greater dementia risk.
***EQ‐5D‐5L*** ^17^— This is a measure of health‐related quality of life (HRQoL), based on information across the five dimensions of mobility, self‐care, usual activities, pain/discomfort, and anxiety/depression. Each dimension is scored on a 5‐point Likert scale (no problems/ slight problems/moderate problems/severe problems/extreme problems). The total score range is 5–25, with higher scores indicating poorer HRQoL.

**TABLE 1:** Summary of tools used for data collection.

Tool name and reference	Purpose/domain	Data collection method
ANU‐ADRI^15^	Dementia risk	Self‐reported
CAIDE^16^	Dementia risk	Self‐reported
Montreal Cognitive Assessment (MoCA)^18^	Cognitive screening	Administered by trained HCP
International Physical Activity Questionnaire (IPAQ) short form^19^	Physical activity	Self‐reported
Centre for Epidemiologic Studies Depression Scale (CES‐D)^20^	Depression symptoms	Self‐reported
EQ‐5D‐5L^17^	Quality of life	Self‐reported
Mediterranean‐DASH Intervention for Neurodegenerative Delay (MIND) questionnaire^21^	Diet	Self‐reported
Australian Type 2 Diabetes Risk Assessment Tool (AUSDRISK)^22^	Diabetes risk	Calculated by using self‐reported data
Absolute cardiovascular disease risk^23^	Cardiovascular risk	Calculated by using self‐reported data
PROMIS satisfaction with participation in discretionary social activities−short form^24^	Social participation	Self‐reported
UCLA 3‐item Loneliness Scale^25^	Loneliness	Self‐reported
Patient Activation Measure (PAM)—Short Form^26^	Patient activation	Self‐reported
Alcohol Use Disorders Identification Test (AUDIT)^27^	Alcohol use	Self‐reported
Charlson Comorbidity Index (CCI)^28^	Comorbidity burden	Extracted from medical data
Tool for Adherence Behaviour Screening (TABS)^29^	Medication adherence	Self‐reported
Self‐assessment of hearing^30^	Hearing	Self‐reported
Self‐Administered Comorbidity Questionnaire (SCQ)^31^	Comorbidities	Self‐reported
STOP‐BANG questionnaire^32^	Sleep apnoea risk	Self‐reported
Insomnia Severity Index (ISI)^33^	Sleep quality	Self‐reported

*Note*: Clinical parameters (height, weight, blood pressure, total cholesterol, high‐density lipoprotein cholesterol [HDL‐C], low‐density lipoprotein cholesterol [LDL‐C], non‐HDL‐C, triglycerides, blood glucose, and glycated hemoglobin A1c) were also collected from participants.

Abbreviations: ANU‐ADRI, Australian National University Alzheimer's Disease Risk Index; CAIDE, Cardiovascular Risk Factors, Aging, and Incidence of Dementia; DASH, Dietary Approaches to Stop Hypertension; PROMIS, Patient‐Reported Outcomes Measurement Information System; UCLA, University of California, Los Angeles.

### 2.2 Risk factors for dementia

Data on a total of 14 risk factors (hypertension, diabetes, hyperlipidemia, depression, physical inactivity, social isolation, current smoking, excessive alcohol intake, low cognitive activity, hearing impairment, family history of dementia, obesity, poor diet, and low education) were collected using validated tools and clinical parameters. Definitions of risk factors and methods of their assessment are provided in Table S1.

### 2.3 Statistical analyses

Data were analyzed using the Statistical Package for Social Sciences (SPSS for Windows Version 30.0. Armonk, NY: IBM Corp.) and R (Version 4.4.0; R Foundation for Statistical Computing, Vienna, Austria). The distributions of continuous variables were assessed using Kolmogorov–Smirnov and Shapiro–Wilk tests. Baseline characteristics of participants were summarized using frequencies, percentages, means (standard deviation), or medians (interquartile ranges). Characteristics and outcomes were compared between sex, age groups, and latent classes using Pearson's chi‐squared test, Independent Samples t‐test, one‐way analysis of variance (ANOVA), Mann–Whitney U‐test, or Kruskal–Wallis test, as appropriate. The Phi correlation coefficient (*φ*) was used to measure the strength and direction of associations between dementia risk factors. A two‐sided *p*‐value < 0.05 was considered statistically significant.

Latent class models were estimated on 14 binary dementia risk‐factor indicators using robust maximum likelihood (MLR) in Mplus version 8.11 (Muthén & Muthén, 1998–2024). Missing data were addressed with full‐information maximum likelihood (FIML) under the missing at random (MAR) assumption, which provides unbiased parameter estimates in the presence of incomplete data.^34^, ^35^, ^36^, ^37^, ^38^ To account for the cluster‐randomized trial design, analyses were specified as TYPE = MIXTURE COMPLEX with CLUSTER = Clinic (31 clinics), yielding cluster‐robust (sandwich) standard errors and adjusted test statistics. All indicators were treated as categorical with a logit link.

Competing models with *k* = 2‐5 classes were estimated using extensive random starts (STARTS = 2000 500; LRTSTARTS = 0 0 500 100), retaining only solutions where the best log‐likelihood was replicated to guard against local maxima. Model selection was guided by a combination of statistical and substantive criteria: the Akaike Information Criterion (AIC), Bayesian Information Criterion (BIC), sample‐size‐adjusted BIC (ssaBIC), entropy, class sizes, and clinical interpretability. The Lo–Mendell–Rubin adjusted likelihood ratio test (LMR‐LRT) was used to compare *k* versus *k*–1 class solution. The Bootstrapped Likelihood Ratio Test (BLRT), although recommended for mixture modelling, is not available under TYPE = COMPLEX in Mplus and was therefore not applied. All models were screened for estimation issues, including boundary thresholds (fixed at ± 15, implying probabilities of 0 or 1 for rare indicators), which may indicate overfitting.

To compare latent classes on continuous distal outcomes, we used the Bolck–Croon–Hagenaars (BCH) three‐step approach.^39^ BCH preserves the latent class measurement model and applies class‐specific weights to adjust for classification error, avoiding bias from assigning the most likely class membership. Overall differences across classes were evaluated with robust Wald *χ* ^2^ tests (df = 2), and pairwise contrasts with robust Wald *χ* ^2^ tests (df = 1), using MLR standard errors and two‐sided *p*‐values.

## 3 RESULTS

A total of 403 participants were recruited into the HAPPI MIND study (62.5% female, mean age 56.4 ± 5.7 years). Most participants were Australian‐born (81.3%) and currently employed (72.3%), with a median Charlson Comorbidity Index (CCI) score of 0.0 (IQR: 0.0 to 1.0) [Table 2].

**TABLE 2:** Characteristics of the study participants (*n* = 403).

Characteristics	Male, *n* = 151	Female, *n* = 252	45−54 years, *n* = 151	55−64 years, *n* = 233	≥65 years, *n* = 19	Total, *n* = 403
Born in Australia^*^	116 (77.3)	210 (83.7)	116 (77.3)	196 (84.5)	14 (73.7)	326 (81.3)
Mainly speaks English at home^†^	141 (94.0)	239 (98.0)	139 (93.3)	223 (98.2)	18 (100)	380 (96.4)
Marital status (living in a relationship)	105 (69.5)	166 (65.9)	104 (68.9)	154 (66.1)	13 (68.4)	271 (67.2)
Currently employed (Yes)^*^	102 (68.5)	188 (74.6)	124 (82.7)	152 (65.5)	14 (73.7)	290 (72.3)
No. of prescribed medicines, median (IQR)^‡^	3.0 [1.0 to 4.0]	2.0 [1.0 to 4.0]	**2.0 [1.0 to 3.0]**	**3.0 [1.0 to 5.0]**	**3.0 [1.0 to 4.0]**	2.0 [1.0 to 4.0]
CCI score, median (IQR)^§^	1.0 [0.0 to 1.0]	0.0 [0.0 to 1.0]	**0.0 [0.0 to 1.0]**	**1.0 [0.0 to 1.25]**	**1.0 [0.0 to 2.0]**	0.0 [0.0 to 1.0]
SCQ total score, median (IQR)^||^	5.0 [2.0 to 9.0]	5.5 [3.0 to 9.0]	**5.0 [2.0 to 8.0]**	**6.0 [3.0 to 9.0]**	**5.0 [3.0 to 9.0]**	5.0 [3.0 to 9.0]
ANU‐ADRI Total, mean (SD)	1.99 (6.73)	1.85 (7.20)	2.99 (6.81)	1.23 (7.15)	1.63 (6.26)	1.91 (7.02)
CAIDE, median (IQR)^¶^	**8.0 [6.0 to 9.0]**	**7.0 [5.0 to 8.0]**	**5.0 [4.0 to 6.0]**	**8.0 [7.0 to 9.0]**	**9.0 [8.0 to 10.0]**	7.0 [5.0 to 9.0]
MoCA, median (IQR)^**^	27.0 [25.0 to 29.0]	27.0 [25.0 to 29.0]	28.0 [26.0 to 29.0]	27.0 [25.0 to 29.0]	27.0 [25.0 to 28.0]	27.0 [25.0 to 29.0]
AUSDRISK score, median (IQR)^††^	**19.0 [13.0 to 23.0]**	**15.0 [12.0 to 19.0]**	**14.0 [11.0 to 19.0]**	**17.0 [13.0 to 21.0]**	**22.0 [16.8 to 24.5]**	16.0 [12.0 to 21.0]
Absolute CVD Risk, median (IQR)^‡‡^	**6.0 [4.0 to 7.0]**	**2.0 [1.0 to 4.0]**	**2.0 [1.0 to 4.0]**	**4.0 [2.0 to 6.0]**	**6.5 [4.0 to 11.0]**	3.0 [2.0 to 6.0]
TABS score, median (IQR)^§§^	**11.0 [6.0 to 14.0]**	**12.0 [8.5 to 14.0]**	11.0 [7.0 to 14.0]	12.0 [8.0 to 14.0]	11.5 [8.5 to 14.0]	12.0 [8.0 to 14.0]
MIND diet score, median (IQR)	**8.0 [7.0 to 9.5]**	**9.0 [7.5 to 10.5]**	9.0 [7.5 to 10.0]	8.5 [7.0 to 10.0]	8.0 [6.5 to 9.5]	8.5 [7.5 to 10.0]
AUDIT score, median (IQR)^‡^	**4.0 [1.0 to 9.0]**	**2.0 [1.0 to 5.0]**	3.0 [1.0 to 7.0]	3.0 [1.0 to 5.0]	3.0 [1.0 to 8.0]	3.0 [1.0 to 6.0]
PAM score, median (IQR)^*^	**66.0 [52.9 to 80.0]**	**72.0 [56.4 to 82.8]**	70.8 [56.4 to 80.0]	68.5 [56.4 to 82.8]	77.5 [49.9 to 91.6]	68.5 [56.4 to 81.4]
EQ‐5D‐5L score, median (IQR)	0.92 [0.83 to 0.96]	0.92 [0.85 to 0.96]	0.92 [0.85 to 0.96]	0.92 [0.82 to 0.96]	0.92 [0.89 to 1.0]	0.92 [0.85 to 0.96]
EQ‐5D‐5L VAS, median (IQR)	70.0 [50.0 to 80.0]	70.0 [50.0 to 80.0]	**68.0 [50.0 to 80.0]**	**71.0 [50.0 to 80.0]**	**80.0 [66.0 to 86.0]**	70.0 [50.0 to 80.0]
PROMIS, median (IQR)^||^	24.0 [19.0 to 30.0]	26.0 [20.0 to 31.0]	**23.5 [18.0 to 29.3]**	**26.7 [20.0 to 31.0]**	**31.0 [23.0 to 35.0]**	25.0 [19.0 to 31.0]
Number of risk factors, median (IQR)	5.0 [4.0 to 6.0]	5.0 [3.0 to 6.0]	5.0 [3.0 to 6.0]	5.0 [4.0 to 6.0]	5.0 [4.0 to 6.0]	5.0 [4.0 to 6.0]
STOP‐BANG score, median (IQR)^||||^	**5.0 [4.0 to 6.0]**	**3.0 [2.0 to 4.0]**	**3.0 [2.0 to 4.0]**	**4.0 [3.0 to 5.3]**	**4.0 [3.5 to 6.3]**	4.0 [2.0 to 5.0]
Insomnia Severity Index (ISI) score, median (IQR)^¶¶^	8.0 [4.0 to 12.8]	8.0 [5.0 to 13.0]	**10.0 [5.0 to 14.0]**	**8.0 [4.0 to 13.0]**	**5.0 [1.5 to 11.0]**	8.0 [4.0 to 13.0]

*Note*: Data are presented as *n* (%) mean (SD) or median (IQR). Bold indicates statistical significance (*p* < 0.05). Higher scores for TABS, MIND Diet, PAM, EQ‐5D‐5L, and PROMIS are indicative of better outcomes, whereas lower scores on all other measures are associated with better outcomes.

Abbreviations: ANU‐ADRI, Australian National University Alzheimer's Disease Risk Index; AUDIT, Alcohol Use Disorders Identification Test; AUSDRISK, Australian Type 2 Diabetes Risk Assessment Tool; CAIDE, Cardiovascular Risk Factors, Aging, and Incidence of Dementia; CCI, Charlson Comorbidity Index; IQR, interquartile range (25th to 75th percentile); MIND, Mediterranean‐DASH Intervention for Neurodegenerative Delay; MoCA, Montreal Cognitive Assessment; PAM, Patient Activation Measure; PROMIS, Patient‐Reported Outcomes Measurement Information System; SCQ, Self‐Administered Comorbidity Questionnaire; TABS, Tool for Adherence Behaviour and Screening.

* Data were missing for 02 participants.

^†^ Data were missing for 09 participants.

^‡^ Data were missing for 06 participants.

^§^ Data were missing for 05 participants.

^||^ Data were missing for 01 participant.

^¶^ Data were missing for 35 participants.

** Data were missing for 15 participants.

^††^ Data were missing for 29 participants.

^‡‡^ Data were missing for 51 participants.

^§§^ Data were missing for 44 participants.

^||||^ Data were missing for 27 participants.

^¶¶^ Data were missing for 14 participants.

Males and females had significantly different scores for CAIDE, AUSDRISK, Absolute CVD Risk, TABS, MIND diet, AUDIT, PAM, and STOP‐BANG (Table 2). Females had higher TABS, MIND diet, and PAM scores, whereas males had higher CAIDE, AUSDRISK, Absolute CVD Risk, AUDIT, and STOP‐BANG scores.

Additionally, significant differences were observed across age groups for CCI, number of prescribed medicines, SCQ, CAIDE, AUSDRISK, Absolute CVD Risk, EQ‐5D‐5L VAS, PROMIS, STOP‐BANG, and ISI (Table 2). ISI score was higher in the 45–54 years group, whereas CCI, number of prescribed medicines, CAIDE, AUSDRISK, Absolute CVD Risk, EQ‐5D‐5L VAS, PROMIS, and STOP‐BANG scores were higher in the older age groups.

The median number of risk factors was 5.0 (IQR: 4.0–6.0), and there were no between‐group differences. The most prevalent risk factors were uncontrolled hyperlipidemia (92.5%), low cognitive activity (72.5%), obesity (57.6%), and hypertension (52.7%) (Table 3). Sex differences in the prevalence of hyperlipidemia, physical inactivity, low cognitive activity, and hearing impairment were observed. Hyperlipidemia (95.3%) and physical inactivity (41.7%) were more common in females, whereas low cognitive activity (78.1%) and hearing impairment (14.7%) were more common in males. Additionally, uncontrolled hyperlipidemia (78.9%) was less prevalent in the older age group, whereas family history of dementia (52.6%) was more common in the older age group (Table 3).

**TABLE 3:** Prevalence of dementia risk factors in Australian middle‐aged adults.

Risk factor	Male, *n* = 151	Female, *n* = 252	45−54 years, *n* = 151	55−64 years, *n* = 233	65 years, *n* = 19	Total, *n* = 403
Uncontrolled hypertension^*^	83 (56.5)	123 (50.4)	67 (46.2)	129 (56.8)	10 (52.6)	206 (52.7)
Uncontrolled diabetes^†^	36 (25.9)	43 (18.2)	27 (19.7)	47 (21.5)	5 (26.3)	79 (21.1)
Uncontrolled hyperlipidemia^‡^	**121 (87.7)**	**225 (95.3)**	**131 (96.3)**	**200 (91.3)**	**15 (78.9)**	346 (92.5)
Depression	59 (39.1)	105 (41.7)	67 (44.4)	90 (38.6)	7 (36.8)	164 (40.7)
Physical inactivity	**48 (31.8)**	**105 (41.7)**	51 (33.8)	94 (40.3)	8 (42.1)	153 (38.0)
Social isolation	21 (13.9)	49 (19.4)	27 (17.9)	43 (18.5)	0 (0.0)	70 (17.4)
Current smoking	29 (19.2)	47 (18.7)	32 (21.2)	41 (17.6)	3 (15.8)	76 (18.9)
Excessive alcohol intake	9 (6.0)	22 (8.7)	12 (7.9)	18 (7.7)	1 (5.3)	31 (7.7)
Low cognitive activity	**118 (78.1)**	**174 (69.0)**	110 (72.8)	167 (71.7)	15 (78.9)	292 (72.5)
Hearing impairment^§^	**22 (14.7)**	**21 (8.4)**	13 (8.7)	27 (11.6)	3 (15.8)	43 (10.7)
Family history of dementia^||^	49 (32.9)	84 (33.7)	**34 (23.0)**	**89 (38.5)**	**10 (52.6)**	133 (33.4)
Obesity	81 (53.6)	151 (59.9)	78 (51.7)	143 (61.4)	11 (57.9)	232 (57.6)
Poor diet	43 (28.5)	55 (21.8)	29 (19.2)	63 (27.0)	6 (31.6)	98 (24.3)
Low level of education	2 (1.3)	3 (1.2)	1 (0.7)	4 (1.7)	0 (0.0)	5 (1.2)

*Note*: Definitions of risk factors and methods of their assessment are provided in Table S1. Bold indicates statistical significance (*p* < 0.05).

* Data were missing for 12 participants.

^†^ Data were missing for 28 participants.

^‡^ Data were missing for 29 participants.

^§^ Data were missing for 02 participants.

^||^ Data were missing for 05 participants.

Table 4 presents the combinations and correlations of dementia risk factors among the study participants. Significant positive correlations were observed between various risk factors/health behaviors: low cognitive activity was significantly associated with depression (*φ = 0.16)*, physical inactivity (*φ = 0.13)*, poor diet (*φ = 0.13)* and smoking (*φ = 0.16)*; obesity was significantly associated with hypertension (*φ = 0.12)*, physical inactivity (*φ = 0.12)*, and diabetes (*φ = 0.16)*; depression was associated with poor diet (*φ = 0.12)*, smoking (*φ = 0.16)*, social isolation (*φ = 0.27)*, and hearing impairment (*φ = 0.11)*; physical inactivity was associated with poor diet (*φ = 0.15)*, smoking (*φ = 0.13)*, and excessive alcohol intake (*φ = 0.12)*; and smoking was associated with poor diet (*φ = 0.16)* and excessive alcohol intake (*φ = 0.22)*.

**TABLE 4:** Combinations and correlations of dementia risk factors among the 403 participants in the HAPPI MIND Trial.

Parameter	Hyperlipidemia^*^	Low cognitive activity	Obesity	Hypertension^†^	Depression	Physical inactivity	Family history of dementia^‡^	Poor diet	Diabetes^§^	Current smoker	Social isolation	Hearing impairment^||^	Excessive alcohol intake
Prevalence of individual risk factors, %	92.5	72.5	57.6	52.7	40.7	38.0	33.4	24.3	21.1	18.9	17.4	10.7	7.7
Prevalence of risk factor combinations and phi coefficient (%, *φ*)													
Low cognitive activity	72.0, −0.06												
Obesity	56.6, −0.098	57.2, −0.01											
Hypertension^†^	52.8, −0.02	**48.8,** −**0.13**	**58.0, 0.12**										
Depression	41.0, 0.03	**45.5, 0.16**	38.4, −0.06	38.3, −0.05									
Physical inactivity	35.5, −0.098	**41.8, 0.13**	**43.1, 0.12**	37.4, 0.01	42.7, 0.08								
Family history of dementia^‡^	34.2, −0.02	32.2, −0.04	33.0, −0.01	36.1, 0.06	31.1, −0.04	32.5, −0.02							
Poor diet	23.7, 0.06	**27.7, 0.13**	25.4, 0.03	21.8, −0.04	**30.5, 0.12**	**32.7, 0.15**	25.6, 0.02						
Diabetes^§^	**18.3,** −**0.23**	19.0, −0.08	**26.7, 0.16**	**27.5, 0.16**	19.5, −0.03	25.4, 0.08	15.1, −0.10	15.9, −0.07					
Current smoking	17.9, 0.03	**22.6, 0.16**	**14.7,** −**0.13**	15.5, −0.07	**26.2, 0.16**	**25.5, 0.13**	18.0, −0.01	**29.6, 0.16**	11.4, −0.09				
Social isolation	17.3, 0.02	18.5, 0.05	15.9, −0.04	18.0, 0.02	**29.9, 0.27**	16.3, −0.02	17.3, −0.00	23.5, 0.09	19.0, 0.02	25.0, 0.097			
Hearing impairment^||^	10.1, −0.04	11.4, 0.03	10.4, 0.01	9.3, −0.03	**14.7, 0.11**	11.2, 0.01	12.0, 0.03	10.4, −0.01	7.6, −0.05	13.3, 0.04	12.9, 0.03		
Excessive alcohol intake	7.8, 0.01	7.9, 0.01	**4.7,** −**0.13**	9.2, 0.05	8.5, 0.03	**11.8, 0.12**	9.8, 0.06	11.2, 0.08	3.8, −0.08	**19.7, 0.22**	5.7, −0.03	9.3, 0.02	

*Note*: Darker shading indicates a higher frequency of risk factor combinations (blue). Bold indicates significant correlation (*p* < 0.05). Results for “Low education” are not presented due to the limited number of participants with this risk factor (*n* = 5).

* Data were missing for 29 participants.

^†^ Data were missing for 12 participants.

^‡^ Data were missing for 05 participants.

^§^ Data were missing for 28 participants.

^||^ Data were missing for 02 participants.

### 3.1 Latent class analysis

Latent class models with two to five classes were estimated and compared using multiple fit indices (AIC, BIC, ssaBIC), entropy, class sizes, and substantive interpretability [Table 5]. Although the five‐class model demonstrated the highest entropy (0.76), it also produced several boundary estimates (i.e., item‐response probabilities fixed at 0 or 1 for rare indicators such as low education and diabetes), suggesting overfitting and reduced generalizability. The four‐class solution showed similar estimation issues and provided no substantively distinct additional profile. The two‐class solution had the lowest BIC but exhibited poor entropy (0.51) and yielded an overly broad “high vs. low risk” division. In contrast, the three‐class model achieved the lowest ssaBIC, acceptable entropy (0.64), clinically meaningful and balanced class sizes, and interpretable risk profiles. On this basis, the three‐class solution was selected as the final model. Estimated class population shares were as follows: Class 1 = 11.4% (*n* = 46); Class 2 = 39.7% (*n* = 160); and Class 3 = 48.9% (*n* = 197). These observed shares were highly consistent with the model‐estimated proportions (10.3%, 40.6%, and 49.1% for Classes 1, 2, and 3, respectively), supporting the stability of class assignment. Classification quality was acceptable to good, with average posterior probabilities of 0.76, 0.83, and 0.83 for Classes 1–3, respectively (Table S2).

**TABLE 5:** Model fit indices for latent class analysis solutions (two to five classes).

No. of classes	Log‐likelihood	Parameters	AIC	BIC	ssaBIC	Entropy	Smallest class (%)	LMR *p*‐value
2	−2695.004	29	5448.0	5564.0	5472.0	0.51	33%	0.24
3	−2667.320	44	5422.6	5598.6	5459.0	0.64	10%	0.61
4	−2648.645	59	5415.3	5651.2	5464.0	0.74	13%	0.46
5	−2632.224	74	5412.4	5708.4	5473.6	0.76	7%	0.79

*Note*: While the five‐class solution yielded the highest entropy, both the four‐ and five‐class models produced boundary estimates (item‐response probabilities fixed at 0 or 1 for rare indicators such as low education and diabetes) and included small classes (< 10%), suggesting overfitting. The three‐class model was retained as the final solution on the basis of the lowest ssaBIC, acceptable entropy, balanced class sizes, and substantive interpretability.

Abbreviations: AIC, Akaike Information Criterion; BIC, Bayesian Information Criterion; ssaBIC, sample‐size adjusted BIC; LMR, Lo–Mendell–Rubin adjusted likelihood ratio test.

**FIGURE 1:**
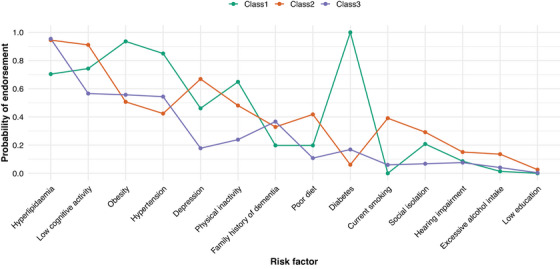
Estimated probabilities of risk factors across latent classes.

Based on estimated probabilities (Figure 1 and Table S3), the three latent classes were defined as follows:

#### 3.1.1 Class 1: High cardiometabolic burden

*Characteristics*: Very high prevalence of obesity (94%), hypertension (85%), and diabetes (100%), along with a high prevalence of hyperlipidemia. This class had negligible smoking (0%) and alcohol use (1%), suggesting that the main risk factors are related to chronic cardiometabolic health conditions.

#### 3.1.2 Class 2: High behavioral and psychosocial risk

*Characteristics*: High prevalence of low cognitive activity (91%) and depression (67%), along with moderate smoking (39%), poor diet (42%), and physical inactivity (48%). This class reflects significant behavioral and psychosocial risk factors, combining lifestyle vulnerabilities with cognitive and mental health burden.

#### 3.1.3 Class 3: Low risk with healthier behaviors

*Characteristics*: Very low probabilities for smoking (6%), social isolation (7%), depression (18%), and alcohol use (4%). With only a moderate prevalence of obesity (56%) and hypertension (54%), this class represents a relatively healthier profile with fewer dementia risk factors and overall better health behaviors.

Pairwise odds‐ratio contrasts (Table S4) highlighted the defining differences between classes. Compared with Class 2, Class 1 had substantially lower odds of hyperlipidemia, low cognitive activity, and poor diet, but markedly higher odds of obesity and hypertension. Relative to Class 3, Class 1 was characterized by higher odds of obesity, hypertension, and physical inactivity, but lower odds of hyperlipidemia. In contrast, Class 2 differed from Class 3 by showing markedly higher odds of depression, smoking, physical inactivity, poor diet, and social isolation, as well as substantially elevated odds of low cognitive activity.

Table 6 indicates distinct outcome profiles across the three latent classes. Class 1 shows the most adverse cardiometabolic profile, with the highest AUSDRISK and absolute CVD risk, while cognitive performance (MoCA) is comparable to the other classes. Dementia risk differs by instrument: CAIDE is highest in Class 1, whereas ANU‐ADRI is highest in Class 2; in both cases, Class 3 has the lowest risk. Class 3 stands out with the highest health‐related quality of life (EQ‐5D‐5L) and the lowest scores on both dementia risk indices, with cardiometabolic risks similar to Class 2. Overall, BCH‐weighted tests confirmed robust between‐class differences for ANU‐ADRI, CAIDE, AUSDRISK, CVD risk, and EQ‐5D‐5L (all *p* ≤.001), but not for MoCA (*p* = 0.395).

**TABLE 6:** Outcome measures across the three latent classes.

Outcome (*n*)	Class 1 Mean (SE)	Class 2 Mean (SE)	Class 3 Mean (SE)	Overall *χ* ^2^ (*p‐*value)	Class 1 vs. Class 2 *χ* ^2^ (*p‐*value)	Class 1 vs. Class 3 *χ* ^2^ (*p‐*value)	Class 2 vs. Class 3 *χ* ^2^ (*p‐*value)
ANU‐ADRI Total (*n* = 403)	6.405 (1.509)	8.624 (0.642)	−4.602 (0.677)	196.897 (< 0.001)	2.988 (0.084)	39.009 (< 0.001)	178.356 (< 0.001)
CAIDE (*n* = 368)	8.091 (0.320)	6.030 (0.167)	5.810 (0.250)	26.328 (< 0.001)	25.440 (< 0.001)	21.157 (< 0.001)	0.548 (0.459)
MOCA (*n* = 388)	26.874 (0.713)	26.531 (0.339)	27.034 (0.323)	1.856 (0.395)	0.218 (0.640)	0.045 (0.832)	1.809 (0.179)
AUSDRISK score (*n* = 374)	25.823 (1.122)	15.613 (0.602)	15.346 (0.817)	56.701 (< 0.001)	56.335 (< 0.001)	39.511 (< 0.001)	0.067 (0.795)
Absolute CVD risk (*n* = 355)	6.772 (0.697)	3.878 (0.308)	4.026 (0.363)	14.849 (0.001)	14.500 (< 0.001)	8.635 (0.003)	0.091 (0.763)
EQ‐5D‐5L score (*n* = 403)	0.783 (0.052)	0.735 (0.022)	0.956 (0.009)	74.628 (< 0.001)	1.190 (0.275)	9.945 (0.002)	69.530 (< 0.001)

Abbreviations: ANU‐ADRI, Australian National University Alzheimer's Disease Risk Index; AUDRISK, Australian Type 2 Diabetes Risk Assessment Tool; CAIDE, Cardiovascular risk factors, aging, and incidence of Dementia; CVD, cardiovascular disease; IQR, interquartile range (25th to 75th percentile); MoCA, Montreal Cognitive Assessment.

## 4 DISCUSSION

This study provided a snapshot of the cognitive function, clinical parameters, QoL, and prevalence of dementia risk factors among middle‐aged Australian adults with two or more potential dementia risk factors recruited from primary care. The study characterized three distinct latent classes that provide a foundation for designing targeted, multidomain interventions consistent with each class's overall dementia risk. By implementing prevention strategies designed to address specific risk profiles, public health and primary care interventions could be more targeted and delivered more efficiently to reduce dementia risk and improve health outcomes. The findings underscore the potential to provide a personalized approach to dementia prevention, particularly within primary care settings, where early identification and intervention for high‐risk individuals may have the greatest impact—but where time and resource implications demand efficient targeting of preventive activities.

Our findings revealed several significant associations among various risk factors associated with cognitive health, suggesting that multiple risk factors often co‐occur, and the pattern of this co‐occurrence is associated with higher risk. The interplay between risk factors highlights the complexity of managing dementia risks and underscores the importance of addressing multiple risk factors simultaneously. Early interventions targeting multiple risk factors concurrently may be more effective in reducing overall dementia risk.^40^, ^41^ Additionally, tailored strategies considering sex and age‐specific risk profiles may further enhance intervention efficiency, targeting more intensive interventions to those at higher risk.

The characterization of three distinct latent classes in at‐risk middle‐aged adults adds novel insights into the complexity of dementia risk profiling. The first class, “High Cardiometabolic Burden,” was marked by a high burden of chronic health conditions, including obesity, hypertension, and diabetes. Still, notably, smoking and excessive alcohol use were absent in this group. This class exhibited the highest risk for CVD and Type 2 diabetes risk (AUSDRISK)—both established dementia risk factors^42^, ^43^—and, correspondingly, the highest CAIDE dementia‐risk score. It should be noted, however, that this association is partly expected given that CAIDE incorporates cardiometabolic risk factors such as hypertension, obesity, and diabetes, which were also indicators in the LCA. Thus, the overlap between class indicators and CAIDE components likely contributes to this finding, and the association should be interpreted cautiously. Evidence suggests that interventions targeting the management of these cardiometabolic conditions, such as lifestyle modification and pharmacological treatment, can effectively reduce CVD risk and may also confer benefits for cognitive health.^44^, ^45^ Furthermore, studies indicate that awareness of dementia risk can motivate individuals differently compared to awareness of CVD risk alone; some patients may be more motivated to engage in preventive behaviors when dementia risk is highlighted,^46^ whereas others respond more strongly to immediate risks such as heart attack or stroke.^47^, ^48^ Therefore, emphasizing the cognitive benefits alongside CVD management could enhance patient engagement and potentially reduce dementia risk in this population.

The second class, “High Behavioural and Psychosocial Risk” exhibited the highest dementia risk (ANU‐ADRI), driven by significant mental health, diet, smoking, and alcohol‐related risk factors. This class had the highest ANU‐ADRI score, which is consistent with evidence linking depression and poor dietary habits to an elevated risk of cognitive decline.^49^, ^50^, ^51^ However, this association may partly reflect shared measurement structure, as ANU‐ADRI incorporates many behavioral and psychosocial factors (e.g., smoking, alcohol, depression) that also define this class. Therefore, the strength of this association should be interpreted with caution. The association between depression and dementia risk is well‐documented, with depression being both a risk factor and a prodrome for cognitive decline.^52^, ^53^ The poor diet observed in this class likely exacerbates the risk, as poor nutrition is a modifiable risk factor for cognitive impairment.^51^ In addition, current smoking and heavy alcohol consumption—both prevalent in this class—are associated with an increased risk of dementia through vascular damage, neuroinflammation, and neurotoxicity.^54^, ^55^

The third class, “Low Risk with Healthy Behaviours,” represents a healthier profile, apart from hyperlipidemia, with fewer risk factors and better health behaviors. Participants in this class had the highest QoL and the lowest dementia risk. This class highlights the potential for lifestyle interventions to reduce dementia risk and promote long‐term cognitive resilience. A combination of healthy behaviors, including regular physical activity, a balanced diet, and mental stimulation, has been consistently shown to lower the risk of cognitive decline.^56^, ^57^ Furthermore, the positive association between healthy behaviors and QoL emphasizes the broader benefits of maintaining good health, not only for cognitive function but also for overall well‐being. From a health system perspective, dementia prevention in this group may be a lower priority, and less intensive interventions, or population‐level interventions, could be appropriate. For instance, low‐resource strategies, such as online tools promoting cognitive health—as demonstrated in the *Maintain Your Brain* trial—can effectively target modifiable risk factors in low‐risk populations.^58^ However, as shown in the Melbourne Diabetes Prevention Study, real‐world interventions in low‐risk individuals may yield modest benefits, likely due to lower baseline risk and behavioral ceilings.^59^ This suggests that, while engaging low‐risk groups remains important, expectations around measurable outcomes should be calibrated accordingly.

These findings have several important implications for dementia prevention strategies, although they should be considered as exploratory and interpreted cautiously given the cross‐sectional study design. The identification of distinct dementia risk profiles underscores the need for personalized, multidomain interventions that target specific combinations of risk factors.

This study has notable strengths. To our knowledge, this was the first known study to specifically examine the dementia risk profiles and correlations of dementia risk factors associated with health and lifestyles among Australian middle‐aged adults (encompassing 14 risk factors). Data were obtained from a large pragmatic cluster randomized controlled trial and were collected by trained nurses using reliable clinical measures and validated scales. Nevertheless, some limitations should be acknowledged. Participants were grouped into age categories to explore potential age‐related patterns. Although the group of 65‐year‐olds was small (*n* = 19), it was included to assess whether distinct patterns might emerge at this transitional age. We acknowledge that the small sample size limits the statistical power and generalizability of findings from this subgroup. Accordingly, results should be interpreted with caution and considered exploratory in nature. The cross‐sectional nature of the study limited the ability to infer causality between the observed associations. Additionally, the overlap between risk factor indicators used in the latent class model and components of ANU‐ADRI and CAIDE indices means that some of the observed associations with dementia risk scores are not entirely independent. These results should therefore be interpreted as exploratory and hypothesis‐generating rather than conclusive evidence of predictive validity. Longitudinal studies are needed to explore possible causal relationships between these risk factors, cognitive decline, and dementia. Further research should investigate the mechanisms underlying the associations identified and explore potential interaction effects between different risk factors. Efforts should also be directed to developing, implementing, and evaluating the effectiveness of tailored interventions for each risk profile.

## 5 CONCLUSION

This study contributes to the growing body of evidence highlighting the multifactorial nature of dementia risk. By characterizing distinct risk profiles and understanding their relationships with cognitive health, cardiometabolic burden, and QoL, this research provides valuable insights for informing early prevention strategies. Future research should explore the effectiveness of tailored interventions for each risk profile and assess their impact on both dementia incidence and long‐term cognitive resilience.

## CONFLICT OF INTEREST STATEMENT

A.J.C. is supported by a National Health and Medical Research Council (NHMRC) emerging leadership 1 grant (APP2009633) and has received grant funding or consulting funding from the Medical Research Future Fund, NHMRC, Dementia Australia Research Foundation and the Pharmaceutical Society of Australia. All funds were paid to the employing institution. A.J.C. also declares she is a national board director for the Pharmaceutical Society of Australia. A.L.B. was supported by an NHMRC Fellowship (APP1135901, 2018‐2022). V.V. is funded by the Rural Health Multidisciplinary Training Program. J.S.B. has received grant funding or consulting funds from the National Health and Medical Research Council, Medical Research Future Fund, Victorian Government Department of Health and Human Services, Dementia Australia Research Foundation, Yulgilbar Foundation, Aged Care Quality and Safety Commission, Dementia Centre for Research Collaboration, Pharmaceutical Society of Australia, GlaxoSmithKline Supported Studies Programme, Amgen and several aged care provider organisations unrelated to this work. All grants and consulting funds were paid to the employing institution. D.T. has received grant funding from GlaxoSmithKline, unrelated to this work and paid to his employer. K.J.A. is funded by ARC Laureate Fellowship (FL19010001). J.G. has received honoraria from GlaxoSmithKline, AstraZeneca and Pfizer for consultancy and educational/research grants from Boehringer‐Ingelheim, GlaxoSmithKline and Pfizer for unrelated projects, all of which have been paid to his employer. All other authors declare no competing interests. Author disclosures are available in the Supporting Information.

## CONSENT STATEMENT

All human subjects provided written informed consent as approved by the Monash University Human Research Ethics Committee.

## Supporting information



Supporting Information

Supporting Information
