# Supplementary material for: Identifying dementia risk profiles for targeted interventions: A latent class analysis of at‐risk middle‐aged Australians
Source: Alzheimers Dement. 2025 Nov 7;21(11):e70888. doi: 10.1002/alz.70888 (PMC12592938; doi:10.1002/alz.70888)
Supplement: Supplementary file 1 — Supporting Information [file ALZ-21-e70888-s001.docx]

# **Supplementary Table 1: Modifiable risk factors, definitions, and methods of assessment**

| **Risk factor** | **Definition of risk factor** | **Method of assessment** |
| --- | --- | --- |
| Hypertension | ≥140 mm Hg systolic and/or ≥90 mm Hg diastolic | Measured by a health professional on more than two occasions in the past 12 months |
| Hyperlipidaemia | >4.0 mmol/L total cholesterol; <1.0 mmol/L HDL-C; >2.0 mmol/L LDL-C; >2.5 mmol/L non-HDL-C; >2.0 mmol/L triglycerides | Laboratory blood test at least once in the past 12 months |
| Type 2 Diabetes | HbA1c >6.5%; fasting glucose ≥7.0 mmol/L; random glucose ≥11.1 mmol/L | Laboratory blood test at least once in the past 12 months |
| Obesity | BMI ≥30 kg/m² | Self-reported or measured by health professional (height and weight) |
| Smoking | Current smoker | Self-report |
| Physical Inactivity | Exercise <30 min/day, <3 times/week | Self-report |
| Poor Diet | MIND diet score <7.5 | Self-report |
| Excessive Alcohol Use | >17 standard drinks/week | Self-report |
| Depression | Centre for Epidemiologic Studies Depression (CESD-10) scale ≥10 score | Health professional assessed or self-reported |
| Social Isolation | Social isolation | Answered "yes" to ≥2 of: lives alone, no weekly contact with family/friends, no weekly social activities, or self-reported loneliness |
| Lack of Cognitive Stimulation | Lack of mentally stimulating activities | Health professional assessed or self-report: fewer than half the days in the past 2 weeks doing cognitive activities (e.g., reading, puzzles, music) |
| Current Hearing Impairment | Suspected or poorly managed hearing impairment | Self-report: "Yes" to trouble hearing on more than half the days in the past 2 weeks |
| Family History of Dementia | Parent or sibling diagnosed with dementia | Self-report |
| Low Education | Less than 8 years of schooling | Self-report |

**Supplementary Table 2. Class membership and classification quality for the three-class solution**

| **Class** | **Model-estimated proportion, (%)** | **Most likely membership, n (%)** | **Mean posterior probability** | **Classification accuracy** |
| --- | --- | --- | --- | --- |
| 1 | 10.3% | 46 (11.4) | 0.76 | Acceptable |
| 2 | 40.6% | 160 (39.7) | 0.83 | Good |
| 3 | 49.1% | 197 (48.9) | 0.83 | Good |

**Note**: Model-estimated proportions are based on posterior probabilities, reflecting expected population shares in each class. Most-likely membership counts represent hard assignment of individuals to their highest-probability class.

**Supplementary Table 3. Item-response probabilities (probability of risk factor = “Yes”) by class**

| **Risk factor** | **Class 1** | **Class 2** | **Class 3** |
| --- | --- | --- | --- |
| Hyperlipidemia | 0.704 | 0.945 | 0.954 |
| Low cognitive activity | 0.743 | 0.911 | 0.566 |
| Obesity | 0.936 | 0.507 | 0.557 |
| Hypertension | 0.850 | 0.424 | 0.544 |
| Depression | 0.461 | 0.669 | 0.178 |
| Physical inactivity | 0.649 | 0.481 | 0.239 |
| Family history of dementia | 0.194 | 0.329 | 0.367 |
| Poor diet | 0.198 | 0.418 | 0.108 |
| Diabetes | 1.000† | 0.061 | 0.169 |
| Smoking | 0.000† | 0.391 | 0.060 |
| Social isolation | 0.208 | 0.292 | 0.068 |
| Hearing impairment | 0.086 | 0.151 | 0.076 |
| Excessive alcohol | 0.014 | 0.136 | 0.041 |
| Low education | 0.000† | 0.026 | 0.004 |

†Boundary estimate (threshold fixed at ±15 by Mplus), implying an estimated class-specific probability of 0 or 1 for this indicator.

**Supplementary Table 4. Pairwise odds ratios (OR, 95% CI) for presence of risk factors across latent classes**

| **Risk factor** | **Class 1 vs 2** | **Class 1 vs 3** | **Class 2 vs 3** |
| --- | --- | --- | --- |
| Hyperlipidaemia | **0.139 (0.033–0.576)** | **0.116 (0.017–0.792)** | 0.835 (0.130–5.357) |
| Low cognitive activity | **0.282 (0.101–0.784)** | 2.209 (0.589–8.286) | **7.846 (3.125–19.701)** |
| Obesity | **14.216 (1.034–195.460)** | 11.610 (0.861–156.652) | 0.817 (0.256–2.606) |
| Hypertension | **7.683 (1.647–35.848)** | **4.750 (1.017–22.185)** | 0.618 (0.304–1.257) |
| Depression | 0.422 (0.094–1.896) | 3.932 (0.354–43.619) | **9.315 (2.028–42.780)** |
| Physical inactivity | 1.992 (0.643–6.170) | **5.863 (1.921–17.897)** | **2.943 (1.331–6.506)** |
| Family history of dementia | 0.493 (0.149–1.626) | 0.416 (0.104–1.667) | 0.845 (0.489–1.460) |
| Poor diet | **0.343 (0.125–0.938)** | 2.034 (0.369–11.223) | **5.935 (1.283–27.461)** |
| Diabetes | Not estimable† | Not estimable† | 0.321 (0.023–4.432) |
| Smoking | 0.000‡ | 0.000‡ | **9.980 (1.909–52.167)** |
| Social isolation | 0.637 (0.260–1.562) | 3.580 (0.689–18.587) | **5.617 (1.394–22.630)** |
| Hearing impairment | 0.525 (0.043–6.473) | 1.146 (0.061–21.504) | 2.184 (0.646–7.380) |
| Excessive alcohol | 0.090 (0.000–69.157) | 0.329 (0.000–301.334) | 3.673 (0.907–14.873) |
| Low education | 0.000‡ | 0.000‡ | 6.796 (0.024–1934.807) |

**Notes:** Bold = statistically significant differences (p < 0.05). OR < 1 = risk factor less common in the first class. OR > 1 = more common in the first class. † “Not estimable” = boundary estimates (probability ≈0 or 1) → ORs undefined/infinite. ‡ “0.000” = near-deterministic separation (risk factor absent/present in entire class). Wide CIs indicate instability due to sparse data or boundary solutions; interpret cautiously.
